# Congenital Afibrinogenemia presenting as antenatal intracranial bleed: a case report

**DOI:** 10.1186/1824-7288-36-1

**Published:** 2010-01-05

**Authors:** Gopakumar Hariharan, Sivji Ramachandran, Rajiv Parapurath

**Affiliations:** 1 Amrita Institute of Medical Sciences (AIMS), Kochi, Kerala, India; 2 Department of Neonatology, Amrita Institute of Medical Sciences (AIMS), Kochi, Kerala, India

## Abstract

Congenital afibrinogenemia is a very rare inherited coagulation disorder, characterized by virtual absence of plasma fibrinogen (factor I). There are only about 250 cases reported in the world literature [1]. We describe a case of congenital afibrinogenemia which presented as an antenatally detected intracranial bleed.

## Case report

**Figure 1:**
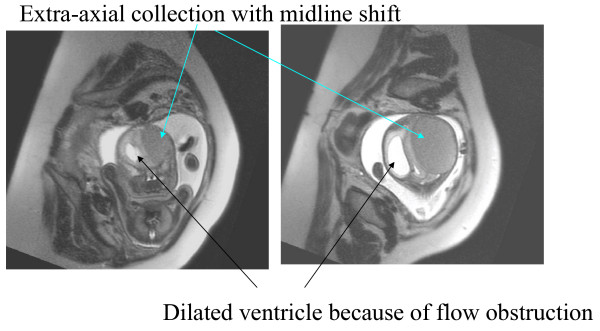
**Fetal MRI, T2 weighted coronal section with a mass effect on the right side with mid-line shift to the left side because of sub-dural bleeding**.

A preterm 29 weeks baby was born of second degree consanguinous marriage to a 28 year old G2A1 mother by elective lower segment caesarean section. Mother was a known case of polycystic ovarian syndrome and was on infertility treatment. Baby was born following intracytoplasmic sperm insertion. She had underwent two intracytoplasmic sperm insertions previously which were both failures. Mother had regular antenatal check ups. Antenatal scan at 29 weeks of gestation showed a large isoechoic intracranial extraaxial collection causing compression of neuroparenchyma and dilatation of the lateral ventricle. A detailed evaluation with a fetal MRI confirmed the finding and showed a fairly large extra axial collection with maximum thickness of 5.7 cms seen on the right with significant mass effect on the ipsilateral cerebral hemisphere, brainstem and cerebellar hemisphere. There was effacement of ipsilateral lateral ventricle and IIIrd ventricle. Significant midline shift was seen with most of the ipsilateral neuroparenchyma pushed towards left causing obstructive hydrocephalus on the left (Fig 1). The collection was homogeneously hyperintense on T1 weighted images. The large extra axial collection suggested hemorrhage on right side with significant mass effect evidenced by the midline shift and hydrocephalus

Baby was born by elective lower segment caesarean section for fetal indication in view of possible uncal coning. Baby was intubated and ventilated at birth due to poor respiratory efforts.

On clinical examination, she had a large head with wide fontanel. Otherwise clinical examination was unremarkable. There was no obvious external congenital malformations. There was no obvious external bleeding tendency.

**Figure 2:**
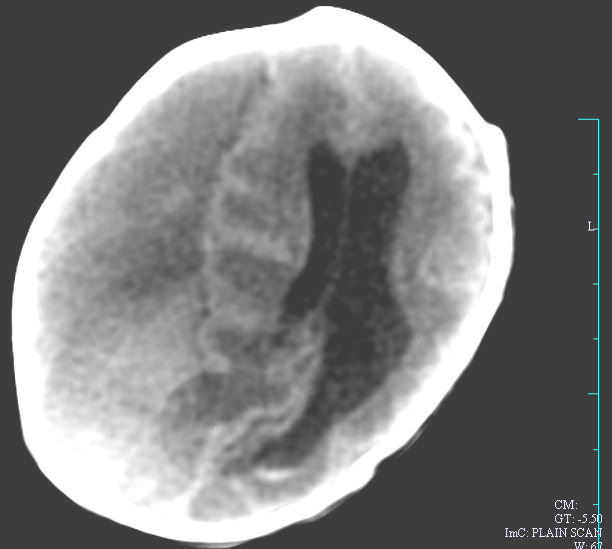
**Plain CT taken postnatally (after draining 50 ml of collected old bleed on day 1)**. The plain CT showing extra-axial collection on Rt.side with a mass effect. The brain architecture is distorted with dilated ventricles.

Neurosonogram done after birth showed a large right sided subdural hemorrhage occupying almost the entire right hemisphere extending to midline with gross shift of midline to left. A Plain CT taken postnatally (after draining 50 ml of collected old bleed on day 1 showed extra-axial collection on Rt.side with a mass effect. The brain architecture was distorted with dilated ventricles (Fig 2)

A detailed blood investigation was done to identify the cause. Hemoglobin done at birth was 8.2 mg/dL. C reactive protein, total count, differential count and micro ESR done did not suggest sepsis. Blood culture was reported sterile. PT and APTT done on second day of life was both significantly prolonged. The initial INR was also prolonged (6.36). Platelet count was 1.3 lakhs/cmm.

The various possibilities for bleeding diathesis were considered and evaluated. Disseminated intravascular coagulation and sepsis was ruled out as sepsis work was reported negative and platelet count was normal. The possibility of a coagulation disorder was considered in view of the clinical profile and abnormal coagulation profile. A fibrinogen level was done to rule out the possibility of afibrinogenemia which was reported to be undetectable. In view of the prolonged INR and APTT along with an undetectable Fibrinogen level, the diagnosis of Congenital afibrinogenemia was made.

Baby was managed with Cryoprecipitates. Packed cell transfusion was given in view of low hemoglobin value initially. The fibrinogen activity was 64 on day 2 of life following one infusion of cryoprecipitate. Values above 100 was achieved by 9 days of life and was maintained so with alternate days of cryoprecipitate infusion. The INR and coagulation profile transiently improved with cryoprecipitate, to subsequently revert to the previous prolonged state when stopped. The decision was made to maintain fibrinogen activity above 100 mg/dL (Normal value - 200 to 400 mg/dL).

2 subdural taps were done on first and third days respectively which drained liquefied blood. Ophthalmology consultation on day 2 of life showed pale disc with pale retina

Baby was ventilated for a total of 14 days and extubated once clinical condition stabilized. Cryoprecipitate was stopped from 22nd day of life to see the magnitude of fall in fibrinogen activity. On 25th day of life, Fibrinogen level was detected to be 45 mg/dL. A repeat neurosonogram on 25th day of life showed persistence of subdural hematoma with pressure effect. There was probably a fresh episode of bleeding associated with significant mass effect. Supportive measures was given including cryoprecipitate with which the child clinically improved.

**Figure 3:**
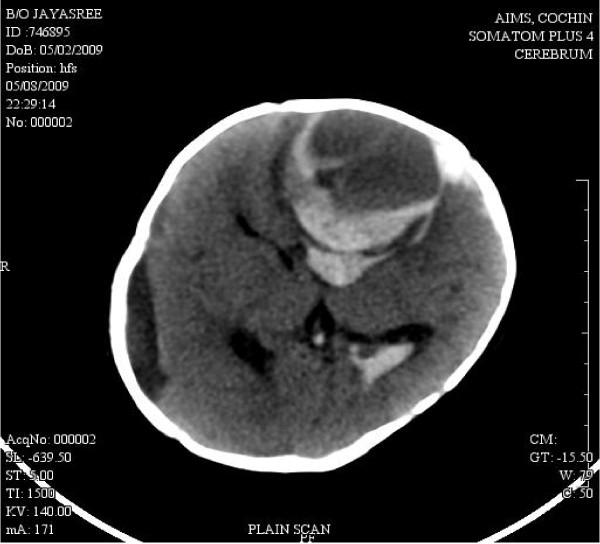
**Acute left frontal bleed (At 6 months corrected age)**.

Child was discharged after the parents were explainted regarding the need for strict neurodevelopmental follow up. Child was relatively asymptomatic until 3 months of corrected age (6 months chronological age) when she was readmitted with one episode of right sided focal seizures along with poor activity and lethargy. Clinical examination showed raised anterior fontanelle tension. Fibrinogen level done at the time of admission was undetectable. Emergency CT scan showed an acute intraparenchymal bleed in left frontal lobe measuring 5 × 4.8 cm (Fig 3). There was another peripheral bleed adjacent to the larger bleed. Intraventricular extension of bleed was also noted with significant midline shift. The subdural hygroma was seen in right convexity with a maximum thickness of 1.2 cm suggestive of residual old subdural bleed. Seizures was controlled with appropriate anticonvulsants. An external ventricular drainage was done to evacuate the clot. She was given supportive measures and cryoprecipitate and was discharged after 3 days of hospitalization. VEP and BERA done at review at 3 months of corrected age was reported normal. Baby had attained social smile at 5 months of corrected age. She had partial head control at 8 months of corrected age. Babbling was present. Child is under strict neurodevelopmental follow up and stimulation programme.

## Discussion

Congenital afibrinogenemia is transmitted by an autosomal recessive gene located on chromosome 4 (q26-q28). Partial deficiency of fibrinogen is called hypofibrinogenemia and is a milder disorder. Afibrinogenemia occurs in the homozygous state while hypofibrinogenemia in heterozygotes [2]. Congenital absence of fibrinogen (Afibrinogenemia) was first described in 1920 and has an estimated incidence of 1: 1-2,000,000 [3].

Congenital afibrinogenemia patients may present with bleeding in the newborn period manifesting as hematomas from traumatic delivery. They may also present with hemetemesis, malena or bleeding from the umbilicus. Other manifestations include bleeding in soft tissues, mucosa and following circumcision. Despite having totally incoagulable blood, these patients normally do not have severe spontaneous bleeding, but the case identification is important to prevent more severe bleeding following injuries or surgery [4]. Intracranial hemorrhage is a rare presentation of the condition in a neonate. Antenatal detection of intracranial bleed is still a rarer entity. Intracranial hemorrhage has been described as a frequent cause of death in this disorder [5-7].

As it is a disorder with the final substrate for formation of the clot missing, results of all screening tests such as clotting time, PT, PTT and thrombin time are abnormal. Platelet functions such as bleeding time, adhesion and aggregation may also be abnormal. Diagnosis is evident by standard fibrinogen assays.

Afibrinogenemic patients have undetectable or nearly undetectable levels of fibrinogen (<10 mg/dL; normal 200-400 mg/dL) by activity-based clotting assays and by measurement of immunoreactive fibrinogen. In the absence of consumptive coagulopathy, an unmeasurable fibrinogen level is diagnostic of the condition [7]. Hemorrhagic symptoms in fibrinogen deficiency are most significant when the plasma level is less than 50 mg/dl [8,9]. Fresh bleeding was noticed in our case, when the fibrinogen fell to 45 mg/dL.

Acute hemorrhagic episodes can be treated with either fresh frozen plasma or cryoprecipitate or fibrinogen concentrate (Cohn fraction I). Each cryoprecipitate bag contains 100 to 150 mg of fibrinogen, and therapy with 100 mg/kg of fibrinogen provides a hemostatic plasma level. The half-life of fibrinogen is 2-4 days and frequent infusions are usually not necessary. However, our patient required frequent transfusions. There was fresh bleeding detected on ultrasonography when cryoprecipitate was stopped temporarily. Recommendations regarding target levels for treating bleeding range from 30-50 mg/dL [10] to 100 mg/dL [11].

Although prophylactic treatment with regular infusions of cryoprecipitate has been advocated by some, [12] it is not recommended by others for several reasons. This is based on the fact that spontaneous bleeding is very rare and mild. Moreover, there is a potential danger of acquiring infections with regular blood product infusion. Antibodies have been reported to form against fibrinogen with resultant thromboembolic complications, particularly pulmonary embolism

Acquired hypofibrinogenemia is common in disseminated intravascular coagulation and primary fibrinolysis. The possibility of acquired afibrinogenemia is unlikely in our case due to its antenatal presentation. A detailed sepsis screen done was unremarkable.

The incidence of spontaneous abortions is high in afibrinogenemic and hypofibrinogenemic females [13-15]. Prepartum and postpartum bleeding are relatively common, and intraabdominal bleeding from ruptured corpus luteal cysts have been reported. We had explained regarding a detailed evaluation of the mother for any Fibrinogen disorders. Maternal Fibrinogen level was reported to be within normal limits. The parents were not willing to do any further genetic work up. We believe that the presence of consanguinity favours the possibility of autosomal recessive inheritance, although a detailed genetic work up was not possible.

Administration of regular infusions of fibrinogen is recommended to maintain pregnancies in afibrinogenemic woman and to reduce the incidence of postpartum hemorrhage [16,17]. Therapy should be initiated as early as possible because fetal loss in the first trimester is frequent. Fetal loss has occurred in some patients with fibrinogen levels above 50 mg/dL as well; therefore, it is recommended that levels be kept above 100 mg/dL through out the duration of the pregnancy.

## Consent

Written informed consent was obtained from the patient for publication of this case report and accompanying images. A copy of of the written consent is available for review by the Editor - in - Chief of this journal

## Competing interests

The authors declare that they have no competing interests.

## Authors' contributions

GK sequenced and drafted the manuscript. SR was involved in the neurodevelopment follow up of the baby. PKR was in charge of the total patient care. All authors read and approved the final manuscript
